# Does Low-Carbon City Construction Improve Total Factor Productivity? Evidence from a Quasi-Natural Experiment in China

**DOI:** 10.3390/ijerph182211974

**Published:** 2021-11-15

**Authors:** Hongfeng Zhang, Lu Huang, Yan Zhu, Hongyun Si, Xu He

**Affiliations:** 1 School of Public Administration and Policy, Shandong University of Finance and Economics, Jinan 250014, China; zhanghongfeng@sdufe.edu.cn (H.Z.); sihongyun@tongji.edu.cn (H.S.); 2 School of Economics, Shandong University of Finance and Economics, Jinan 250014, China; yanshanhexu@126.com

**Keywords:** low-carbon city, total factor productivity, difference-in-differences, quasi-natural experiment

## Abstract

Low-carbon city construction (LCC) is an important strategy for countries desiring to improve environmental quality, realize cleaner production, and achieve sustainable development. Low-carbon cities have attracted widespread attention for their attempts to coordinate the relationship between environmental protection and economic development. Using the panel data from 2006 to 2017 of prefecture-level cities in China, this study applied the difference-in-differences (DID) method to analyze the effects of LCC on the total factor productivity (TFP) of the cities and its possible transmission mechanism. The results show significantly positive effects on TFP, but the effects on each component of TFP are different. Although the LCC has promoted technical progress and scale efficiency, it has inhibited technical efficiency. The accuracy of the results has been confirmed by several robustness tests. Mechanism analysis showed that the pilot policy of low-carbon cities has promoted technical progress and scale efficiency by technological innovation and the upgrading of industrial structure, but resource mismatches among enterprises have been the main reason for reduced technical efficiency. Regional heterogeneity analysis showed that the effects on TFP in the eastern region have been more significant than in the central and western regions. In the eastern region, they have promoted technical progress, while in the central and western regions, they have promoted technical progress and scale efficiency but hindered technical efficiency. This paper presents our findings for the effects of LCC on economic development and provides insightful policy implications for the improvement of technical efficiency in low-carbon cities.

## 1. Introduction

In recent years, global climate change has become severe and the ecological environment has experienced frequent crises [1]. Carbon emissions have become a key source of global warming and the construction of low-carbon cities has gradually become an important step toward curbing global warming and improving environmental quality. Only by improving air quality can human health and social welfare be guaranteed [2]. The key to building a low-carbon city with low emissions and high energy efficiency is to handle the relationships among energy consumption, environmental protection, and economic development properly [3].

According to the Environmental Performance Index (EPI) 2020 of 180 countries, China’s environmental performance ranks 120th and lags behind Russia, South Africa, and Brazil [4]. In 2018, China was the largest emitter of carbon and accounted for 28% of global emissions [5]. The country has also experienced numerous serious incidents of environmental pollution [6] and has faced tremendous pressure from the international community [1]. China has made great efforts in improving resource utilization efficiency, developing renewable energy, and reducing carbon emissions and air pollution [7], but its environmental performance still lags far behind that of many developed countries.

The social benefits of environmental regulations such as policies for low-emission zones are higher than the cost of governance, which is an important way to achieve a win-win situation between environmental improvement and economic development [8]. The construction of low-carbon cities is a dual strategy to cope with global climate change and high-quality economic development [9]. The pressure of the international community, prominent domestic environmental problems, and the demand for the upgrading of the energy industry has motivated the development of low-carbon cities [10]. Therefore, the Chinese government approved the establishment of three groups of pilot low-carbon cities during 2010–2017 to explore which mode of development would be suitable to China’s national conditions [11].

Total factor productivity (TFP) is the main driving force of national economic growth. As a measure of technological change, it has a profound effect on national production capacity [12]. Balancing environmental protection and economic development has become a crucial issue that raises the question. As an important regulatory policy to improve environmental quality, does low-carbon city construction (LCC) improve TFP to achieve a win-win situation between environmental protection and economic development? Some scholars have tried to answer this question. Cheng et al. used a difference-in-differences (DID) model with the panel data of prefecture-level cities from 2007 to 2016 and found that a low-carbon pilot policy had had significant, positive technological and structural effects on urban green total factor productivity (GTFP) [3]. The GTFP, as a green growth indicator, considers not only the economic growth but also the constraints of the environment and energy. Qiu et al. believed that a low-carbon pilot policy promotes GTFP through technological innovation, resource allocation, and energy intensity [13]. The goal of LCC is not only to promote green development but also to achieve the long-term sustainable development of urban economies. Having studied the relationship between a low-carbon pilot policy and the TFP of listed enterprises, some scholars have found that the construction of low-carbon cities was conducive to the improvement of the TFP of enterprises with technological progress and the optimal allocation of resources as the main mechanisms [14]. However, the effects of a low-carbon pilot policy on overall regional TFP have not yet been explored. Few studies have decomposed regional TFP and its impact mechanism. The exploration of how a low-carbon pilot policy contributes to long-term urban development has both theoretical and practical significance for the construction of low-carbon cities, as well as the realization of cleaner urban production and sustainable economic development, in China. To provide recommendations to the departments responsible for policymaking and implementation that promote the sustainable development of China’s economy, this study focused on the effects of the low-carbon city policy on productivity growth and examined if the construction of the pilot cities had achieved improvements in environmental quality at the expense of economic development.

This study applied a DID model to an empirical analysis of the effects of the LCC on the TFP of prefecture-level cities and its components, then explored the robustness and regional heterogeneity of the benchmark estimations and analyzed the effect mechanism of the LCC on TFP. The following are the innovative aspects and contributions of this study. First, new evidence was uncovered by the DID method, thus allowing for testing Porter’s hypothesis and effectively solving the endogenous problems commonly faced by evaluations of policy effects. Second, a theoretical basis for measures that improve production efficiency was established by the decomposition and analysis of the differential changes in the three components of the TFP of various Chinese cities. Third, this study has contributed to the research on the cause-effect relationship between the LCC and TFP, as well as provided recommendations for the LCC and economic sustainable development of developing countries, by the measurement and analysis of the changes in the upgrading of urban industrial structures, levels of technological innovation, and resource allocation efficiency.

## 2. Literature Review and Policy Background

### 2.1. Literature Review

Proposed by Solow [15], the theory of TFP has been regarded as a comprehensive indicator of technological progress, the upgrading of industrial structures and management modes, and the improvement of production quality [16]. Additionally, it has been widely used as a comprehensive measure of social production efficiency in research on the sources of economic growth and sustainable economic development [17]. The existing literature has focused on macro-TFP [18,19] and micro-TFP [20,21,22], as well as factors that affect TFP, such as capital accumulation [23], R and D and innovation in technology [24,25], international trade and globalization [26], and economic shocks [27,28].

With many scholars in various countries, the current research on the effects of environmental regulations in developed countries is relatively rich. Chay and Greenstone evaluated the health effects of the 1970 Clean Air Act Amendments (CAAA) in the United States and found that they had suppressed air pollution and produced greater economic benefits [29]. From a long-term perspective, the CAAA has played an important role in controlling haze [30]. The environmental regulations of developing countries have also been discussed by some scholars. Greenstone and Hanna found that the effects of environmental regulatory policies implemented in India were different and that public support was the key factor in determining the effects [31]. Tanaka pointed out that the policy of the “Two Control Areas” implemented in China has significantly reduced infant mortality and has brought high health benefits [32]. The carbon emission trading system in China has promoted green total factor productivity [33]. In addition, studies have shown that environmental regulations could effectively improve regional air quality [9,34] while promoting productivity [35].

The development of low-carbon cities is an important environmental regulatory measure. Several studies have examined environmental improvement, costs-benefits, and social welfare in such cities in various countries. Ellison et al. found that the low-emission zone implemented in London played an important role in improving air quality [36]. The low-carbon zones implemented in Germany have significantly reduced air pollution and achieved health benefits greater than the cost of upgrading [8]. However, some scholars believe that the low-carbon zones have improved air quality, but the improvement was weak and did not promote the health of infants [37]. China has subsequently implemented a pilot low-carbon city policy, which has achieved the goal of improving the carbon emission efficiency of cities and has created certain spatial spillover effects [9,38]. Not only has the policy had significantly positive effects on industrial structure supererogation [11] but has also promoted the economic transformation and green development of low-carbon cities through technological innovation, energy structure optimization, and carbon sequestration [3,13].

To sum up, the current research on low-carbon cities is relatively rich, but few studies have evaluated their effects on the TFP of the cities. This study explored the effects of China’s pilot LCC on TFP and its components. Knowledge of the effects is of great significance to achieving a win-win scenario for both economic development and environmental protection.

### 2.2. Policy Background

The main cause of global warming is carbon emissions caused by human activities [11], which has become a key problem that all human beings should urgently solve. The carbon emissions of China increased by an average of 2.2% annually from 2009 to 2018. Therefore, China is under tremendous pressure from the international community [5]. In 2021, the Chinese government announced the goal of achieving peak carbon dioxide emissions by 2030 and carbon neutrality by 2060. Cities are an important driver of energy use and related carbon emissions [39]. As an important part of the measures to address climate change in China, the LCC is an exploration of a development model that decouples economic growth from carbon emissions by selecting regions with different types, resource endowments, and development stages for pilot projects [40]. In 2010, the National Development and Reform Commission (NDRC) issued a notice on pilot projects for low-carbon provinces and cities. Thus, three groups of pilot low-carbon provinces and cities were launched. In 2010, the first group of pilot low-carbon cities was implemented in the provinces of Liaoning, Yunnan, Guangdong, Shanxi, and Hubei, as well as in the cities of Tianjin, Guiyang, Chongqing, Xiamen, Shenzhen, Nanchang, Hangzhou, and Baoding. In 2012, the NDRC designated 1 more province and 28 more cities as the second group, then, in 2017, designated 45 more cities as the third group. Therefore, 81 pilot low-carbon cities covering 31 provincial administrative regions are currently in operation.

The notice required each pilot city to formulate low-carbon development plans and supporting policies for developing low-carbon industries, establishing carbon dioxide emission management systems, encouraging low-carbon consumption and lifestyles, and exploring a regional low-carbon development model suitable to the city’s particular geographical conditions, resource endowments, and economic foundation [9]. Each city’s local government sets the carbon emission and energy intensity targets under the national benchmark. The pilot policy was to implement a reporting and approval system, which is a development model that combines top-level design with pilot demonstrations. Pilot low-carbon cities are not only experiments for testing the effects of environmental regulations but also pioneers that explore cleaner production and sustainable development.

The construction of pilot low-carbon cities is closely related to economic and social development, so they have affected production efficiency and economic development in the following ways. First, the construction has promoted the adjustments of the industrial and energy structures because carbon emission efficiency has been improved by the development of green and low-carbon industries, as well as the transformation of high-carbon industries. The adjustments require not only raising the market access threshold and carbon emission standards for high-polluting industries but also applying low-carbon technologies to optimize and upgrade traditional industries. Second, the construction has also promoted the reallocation of resources [14]. Policy trends cause more resources to flow into the low-carbon environmental protection industries, leading to the excessive concentration of production factors in these industries and possibly influencing the coordinated development of these industries and the optimal allocation of resources. Finally, construction has stimulated corporate innovation. As a result of the increase in pollution control and production costs, companies would increase their R and D investments to improve their levels of technological innovation and production efficiency [1], as well as accelerate the introduction of cleaner and renewable sources of energy, low-carbon transportation, low-carbon buildings, and other emerging technologies, thereby promoting patent flows and innovation spillovers.

## 3. Methodology and Data

### 3.1. Model Description

**Figure 1:**
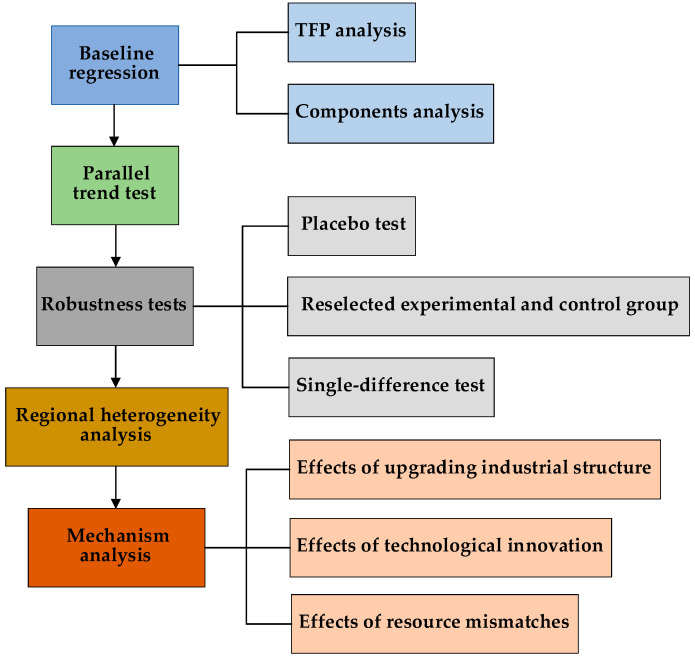
Research framework and process. TFP: the total factor productivity.

The pilot low-carbon cities provided an appropriate quasi-natural experiment, so they constituted the experimental group while the remaining cities constituted the control group. The DID method was used to analyze the effects of the LCC on TFP and its components. The research framework of this paper is illustrated in Figure 1.

The pilot cities of the first group in 2010 were small and mostly situated in non-representative provinces. The period for the implementation of the third group in 2017 was relatively short and the relevant data statistics were lacking. To ensure the accuracy of the evaluation, only the effects of the second group in 2012 were analyzed. To avoid the first group’s interference with the estimations, the prefecture-level cities corresponding to the first group were excluded. As limited data were available, 184 prefecture-level cities for 2006 to 2017 were selected as the sample. Finally, the following DID model for two-way fixed effects was formulated:

(1) $$TFP_{i,t}=\beta_{0}+\beta_{1}Policy_{i,t}+\sum_{k=1}^{n}\beta_{k}Control_{k,i,t}+\gamma_{t}+\mu_{i}+\varepsilon_{i,t}$$

where the dependent variable is $TFP_{i,t}$ while the subscripts i and t represent the i-th city and t-th year, respectively. The core explanatory variable is $Policy_{i,t}$, which is the pilot low-carbon policy. $Control_{k,i,t}$ is a series of control variables that include industrial structure, level of economic development, degree of openness, population density, government size, fiscal autonomy, and government R and D funding intensity. $\gamma_{t}$ represents the time fixed effect, which controls the characteristics of the time level and does not change with regional changes, $\mu_{i}$ represents the each prefecture-level city’s regional fixed effect, which controls the factors that do not change over time, and $\varepsilon_{i,t}$ is the random error term. $\beta_{1}$ is the core estimation parameter, which represents the effects of the policy on the TFP of the cities. If $\beta_{1}$ is positive, then the policy has been conducive to the improvement of the TFP; otherwise, it has had inhibitory effects.

We decomposed TFP into scale efficiency, technical progress, and technical efficiency, then incorporated enterprise control variables including enterprise scale, financial situation, return on assets, proportion of intangible assets, and government subsidy intensity into the mechanism analysis. The specific variable settings are described in detail later.

### 3.2. Data and Variables

The focus of this paper is the effects of LCC on TFP and a detailed analysis of the robustness, regional heterogeneity, and mechanism of these effects. In addition to policy implementation, many factors affect TFP, so many control variables were added. The concrete methods for variable selection and processing are presented in Table 1.

#### 3.2.1. Dependent Variable

The core dependent variable is the TFP of the cities. The three most common methods to measure TFP are the solow residual method (SRM), stochastic frontier analysis (SFA), and data envelopment analysis (DEA). Compared with other methods, the TFP calculated based on SFA can better reflect its authenticity. The production function model in the form of transcendental logarithm relaxes the constant substitution elasticity hypothesis and can test the effectiveness of the function form, thereby ensuring a better fitting effect. In this paper, the calculation method of TFP refers to that of [41]. By SFA and the production function of a transcendental logarithm, TFP could be decomposed [42]. Specifically, we took the derivative of the production function of a transcendental logarithm with respect to time and then decomposed it into scale efficiency, technical progress, and technical efficiency. The output index is expressed as the gross domestic product (GDP). Because the nominal GDP cannot reflect the actual output level accurately, this study used the GDP deflator index of each province to adjust the GDP of each city with the prices in 2006 as the base. The input indicators are labor and capital. The proxy variable of the labor input is the total number of employees in each city, which is expressed by the sum of private employees and unit employees in the China City Statistical Yearbook. The capital stock is calculated by the method of perpetual inventory, which involves three important indexes: the capital stock in the base period, the price index, and the depreciation rate. The earlier the base period of the capital stock, the more accurate is the calculation of the subsequent capital stock. The base period 1991 was chosen according to the availability of data. The capital stock at the city level in the base period was converted from provincial-level to municipal-level capital stock according to the proportion of each city out of the total social fixed asset investment of each province in 1991. The data of Zhang were used for the base period capital stock at the provincial level [43]. According to the fixed assets investment price index of the province in which each city was located, fixed assets investment was deflated with the prices in 2006 as the base. A depreciation rate of 9.6% was used [43]. The data were taken from the China Statistical Yearbook, China City Statistical Yearbook, and China Economic Database (CEIC).

#### 3.2.2. Key Explanatory Variable

The explanatory variable is the dummy variable of the pilot low-carbon policy. If a city was a pilot low-carbon one in 2012, then the dummy variable is set to 1; otherwise, it is set to 0. Because of the pilot policy in Hainan Province and the lack of data in some areas, 1 province and 28 cities that piloted low-carbon cities in 2012 actually corresponded to 26 prefecture-level cities through screening and matching. That means there were 26 cities with low-carbon policy, and 158 non-low-carbon policy cities used in the research. The data were taken from the notice of the second group of low-carbon pilot provinces and cities issued by the NDRC in December 2012.

#### 3.2.3. Control Variables

To control the influences of other factors on TFP, a series of control variables were selected from the relevant literature. Some studies have found that industrial structure and openness to international trade are both important factors affecting TFP [38]. Therefore, we used the proportion of the added value of the secondary industry in GDP to express the industrial structure and the ratio of the actual foreign direct investment (FDI) and GDP to express the degree of openness. The data of the actual FDI were in US dollars, so currency conversions were based on the intermediate exchange rate of each year. Improvements in TFP were generally accompanied by increases in economic development. The ratio of real GDP after deflation based on the 2006 base period to the total population at the end of the year indicated the level of economic development. The ratio of the revenue to the budgeted government expenditures was used to express fiscal autonomy. The new theory of economic growth postulates R&D as an important factor affecting innovation output and technological innovation with important effects on TFP [44]. Since the data on R&D expenditures were not available for each city, the sum of government fiscal expenditures on science and education was used as a proxy variable for government R&D funding intensity [45]. In addition, the ratio of the total population to the administrative area at the end of the year and the ratio of the government budget expenditure to the regional GDP were used to represent population density and government size, respectively, to control the effects of the degrees of population agglomeration and government intervention, respectively, on TFP.

Enterprise-level control variables were incorporated into the mechanism analysis. The total assets of the enterprise represent the enterprise scale. The ratio of total liabilities to total assets indicates the level of financial risk. The ratio of net profit to total assets represents the return on assets. The proportion of net intangible assets to total assets represents the proportion of intangible assets. The ratio of government subsidies to operating income represents the government subsidy intensity [18,46]. The data were taken from the China Statistical Yearbook, China City Statistical Yearbook, CEIC, and China Stock Market and Accounting Research Database (CSMAR). In this study, some variables were processed logarithmically. Table 2 presents descriptive statistics of the results for each variable.

## 4. Empirical Analysis

### 4.1. Baseline Regression

Equation (1) was used in the DID model to estimate the effects of LCC on TFP. The baseline estimations are shown in Table 3. Columns (1) to (8) were gradually added to the control variables and the winsorized 1% at the top and bottom were adopted. The estimated coefficients are significant and positive, indicating that the establishment of the pilot low-carbon cities had significantly promoted their TFP. Column (9) shows the estimated results after data from the most economically developed cities, such as Beijing, Shanghai, Guangzhou, and Wuhan, had been excluded to avoid the special effects due to their higher levels of economic development. The estimated coefficient of the LCC is still significant at a 5% level.

Table 3 shows that the pilot low-carbon city policy had significantly positive effects on TFP. To investigate the causes of these effects, TFP was decomposed and the effects of the LCC on the components of TFP were estimated. Columns (1) and (2) in Table 4 show that the effects on scale efficiency are positive and significant, indicating that the pilot cities have played positive roles in promoting regional industrial upgrading and specialization, as well as realizing economies of scale, as the result of the preferential policies and incentive measures implemented by the policy. Columns (3) and (4) show that the effects on technical progress are positive and significant, indicating that the cities have raised their levels of urban technological progress. The policy was accompanied by environmental protection standards and performance assessment. On the one hand, the cities reduced the number of enterprises with high emissions, high energy consumption, and high pollution. On the other hand, they encouraged urban technical progress by engaging in green technology innovation and achieved the goals of energy-saving and emission reduction with new equipment, technology, and methods. Columns (5) and (6) show that the effects on technical efficiency are negative and significant, indicating that the cities have ignored the internal relationships between the enterprises and the reasonable allocation of resources after the implementation of the policy. This lack of attention has led to the misallocation of resources, as well as has significantly and negatively affected urban technical efficiency.

### 4.2. Parallel Trend Test

**Figure 2:**
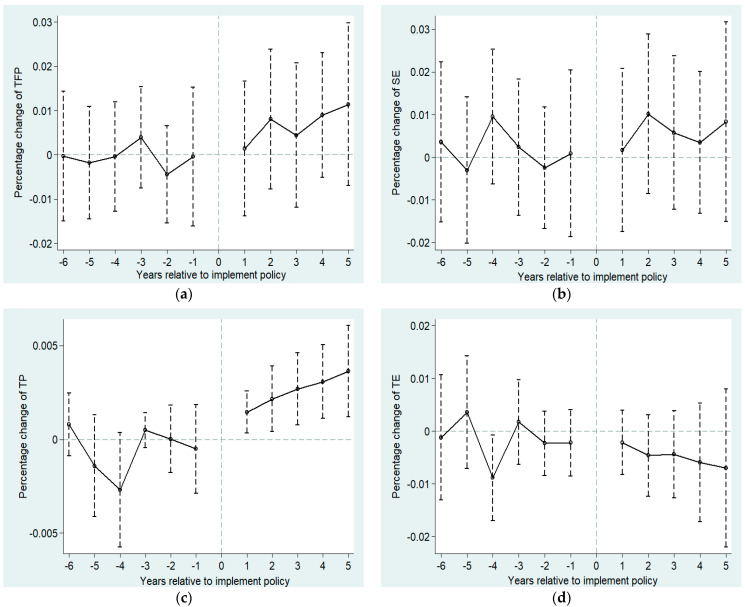
Parallel trend test. The horizontal axis indicates the years before and after the policy’s implementation. The vertical axis represents the percentage change in each variable of the experimental and control groups. (**a**–**d**) describe the percentage changes in TFP, SE, TP and TE differences between the experimental group and the control group, respectively.

The parallel trend hypothesis of the DID method assumes no time trend difference between the experimental and control groups before the policy implementation. Therefore, an examination was required of whether or not a time trend difference in TFP existed between both groups before the establishment of the pilot cities. Referring to Beck et al. [47], we designed a parallel trend test with the formula:

(2) $$TFP_{i,t}=\beta_{0}+\beta_{1}Policy_{s,t}^{-6}+\beta_{2}Policy_{s,t}^{-5}+\cdots +\beta_{10}Policy_{s,t}^{+4}+\beta_{11}Policy_{s,t}^{+5}+\sum_{k=1}^{n}\beta_{k}Control_{k,i,t}+\gamma_{t}+\mu_{i}+\varepsilon_{i,t}$$

where $Policy_{s,t}^{-j}$ and $Policy_{s,t}^{+j}$ are the respective dummy variables in the j-th years before and after the policy implementation. We excluded the years of the implementation, estimated the dynamic changes in the differences of the effects of the LCC on TFP between the experimental and control groups in the sample period, and drew the parallel trend test chart shown in Figure 2. Before the establishment of the cities, no significant differences in TFP and its components for both groups were present. After the implementation, the effects and differences gradually appear, thus verifying the parallel trend hypothesis.

### 4.3. Robustness Tests

#### 4.3.1. Placebo Test

The placebo test is a robustness test that changes the timing of policy shocks artificially [48]. We assumed that the improvement in TFP had not been due to the pilot policies but to the economic and social development over time, which are not related to the cities. To eliminate the influence of conventional random factors for a placebo test, we referred to [49] to set the false year of the implementation of the policy. We advanced the implementation times of the real policy by one and two years, then estimated the effects on TFP and its components. Columns (1–4) and (5–8) in Table 5 show the results after one and two years, respectively. The estimated coefficients are not significant; therefore, the above assumption is disconfirmed, whereas the effects of the LCC on TFP are confirmed not to be the result of unobservable factors.

#### 4.3.2. Reselected Experimental and Control Groups

For the robustness test of whether the effects applied only to the second group, we added the first group as the experimental group to the sample while treating the other cities as the control group. Table 6 shows that the results are the same as those of the baseline regression, which are more robust. Hence, TFP and its components had not been affected by the chosen experimental group.

#### 4.3.3. Single-Difference Test

Following the traditional approach, we used the single-difference test method to estimate the effects of the LCC on the TFP of the pilot cities. This approach requires controlling only other variables and regional fixed effects, and then carries out regression estimation [50]. The results are shown in Table 7. Regardless of the addition of the control variables, the pilot cities have significantly and negatively affected TFP and technical progress, indicating that this method has not solved the problem of missing variables that change over time. These missing variables have caused the sign of the coefficient to change, thus skewing the accuracy of the results. In addition, the estimated coefficients of scale and technical efficiency are significantly higher than those in the baseline regression, indicating that the method has overestimated the effects because of the uncontrolled time change trend. The conclusion reached by the DID method is more reliable.

### 4.4. Regional Heterogeneity Analysis

The per capita carbon dioxide emissions of China are not evenly distributed over its vast territory [51]. This unbalanced spatial distribution is the result of differences in economic and social development, energy structure, and productivity growth rates. The formulation and implementation of various environmental regulatory policies are also different over various regions. Therefore, the heterogeneous effects of the LCC on the TFP and its components of each region have to be considered, so we divided the full sample into the eastern developed region and into the central and western underdeveloped regions. The results are shown in Table 8.

The LCC has significantly and positively affected the TFP of the eastern region. Specifically, the pilot cities have significantly and positively affected technical progress but not significantly affected scale and technical efficiency. The regression estimations for the central and western regions are basically the same as that for the full sample. The pilot cities have significantly promoted scale efficiency and technical progress but have inhibited technical efficiency. In sum, the pilot cities have not had significant effects on the TFP of the central and western regions.

### 4.5. Mechanism Analysis

**Figure 3:**
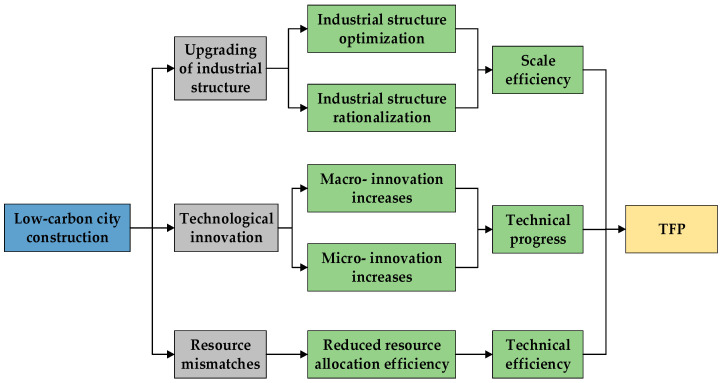
Mechanism of effects of low-carbon city construction (LCC) on the total factor productivity (TFP).

Section 4.1, Section 4.2, Section 4.3, Section 4.4 and Section 4.5 have shown that the LCC has positively affected TFP. The next question to answer is, “What is the transmission mechanism of these effects?” This section discusses our testing of the internal mechanism and the pathways of how the LCC has affected the scale efficiency, technical progress, and technical efficiency of the cities (as shown in Figure 3).

#### 4.5.1. Effects of Upgrading Industrial Structure

The LCC has promoted the development of the tertiary and low-carbon environmental protection industries so that they could facilitate the upgrading and optimization of the industrial structures [11]. On the one hand, the optimization of industrial structure is conducive to the transitioning of the primary and secondary industries to the tertiary industry. On the other hand, the rationalization of industrial structure is conducive to promoting the specialization of the division of industrial labor, the scale of production, and the coordinated development of industries, thereby promoting the realization of scale economies and the improvement of scale efficiency.

Following Gan et al. [52], we adopted the optimization and rationalization of the industrial structure to measure its upgrading. The industrial structure optimization means that the proportion of the added value of the primary industry in the national economy is gradually reduced, while the proportion of the added value of the secondary and tertiary industries is gradually increasing. The industrial structure rationalization means that the industries with higher added value in the three industries also have more human capital investment. We used the ratio of the added value of the tertiary to the secondary industry to measure the level of optimization (TS) and the industrial structure Theil index to measure the level of rationalization (TL):

(3) $$TL_{i,t}=\sum_{m=1}^{3}y_{i,m,t}\times \ln\left(y_{i,m,t}/l_{i,m,t}\right),m=1,2,3$$

where $y_{i,m,t}$ represents the proportion of the added value of industry m in region i in year t out of the GDP and $l_{i,m,t}$ represents the proportion of employees in industry m in region i in year t out of the total number of employees. The value of TL is inversely proportional to the degree of rationalization. A value of 0 indicates that the industrial structure is in equilibrium [53]. The regression estimations of the LCC on upgrading are shown in Table 9. After the time and the regional fixed effects are added, the standard errors become clustered at the city level. After the control variables have been added in sequence, the estimated coefficients of the LCC in all the columns, except for Columns (5) and (6), are significant, indicating that the pilot low-carbon policy had significantly optimized and rationalized the industrial structure.

#### 4.5.2. Effects of Technological Innovation

The effects of technological innovation are reflected at both the macro- and micro-levels. At the macro-level, the number of patents granted by prefecture-level cities is affected by the pilot low-carbon policy. The micro-level is represented by the effects of the policy on the number of patents granted to enterprises in prefecture-level cities.

The indicators for measuring technological innovation are also divided into macro- and micro-indicators. The number of patents granted per 10,000 people for total patent (Innovation), invention patent (Invention), utility models patent (Utility Models), and design patent (Design) was used to measure the capabilities of the cities for technological innovation. Because of the time of the policy’s implementation and the availability of relevant enterprise data, this study used the number of total patents and various types of authorizations for patent applications by the listed companies in each city as proxy variables. Then, we further divided the sample of listed companies into Manufacturing and Non-manufacturing samples for a regression of the effects of the LCC on technological innovation at the macro-level. The results are shown in Table 10. The estimated coefficients of the LCC are all significantly positive, indicating that the policy has promoted the technological innovation of the cities by increasing the total number of urban patent applications for inventions, utility models, and designs.

The regressions at the micro-level of the effects of the LCC on the technological innovation of the listed companies are shown in Table 11. The estimated coefficients are not significant for the full sample. However, they are significantly positive for the manufacturing companies but significantly negative for the non-manufacturing ones, indicating that the pilot cities have significantly promoted the technological innovation of the manufacturing companies but inhibited that of the non-manufacturing ones. To investigate the reasons for these results, which are shown in Table 12, we estimated the effects of the LCC on the R&D expenditures. Again, the estimated coefficients are not significant for the full sample but are significantly positive for manufacturing while being significantly negative for non-manufacturing. These results indicate that the pilot cities have significantly restrained the R&D expenditures of the non-manufacturing companies, explaining, to a certain extent, the relative decrease in the number of patents granted to them.

Table 13 shows the regression estimations of the effects of the LCC on various types of patent applications by all the listed companies, indicating that the LCC has significantly inhibited the increase in the number of patent applications for inventions but has increased the number of applications for the utility models and design patents. The effects of the LCC on the number of patents granted have been manifested in the significantly positive effects on the inventions, utility models, and design patents of the listed manufacturing companies. The inhibitory effects of the LCC on the number of patents granted to listed non-manufacturing companies are mainly reflected in the reduction in the number of invention patents granted, and the increase in the number of design patent applications by listed non-manufacturing companies. No significant effects have been found for utility model patents.

The positive effects of the LCC on the patent applications of listed companies have been manifested mainly in the promotion of the patent applications of the manufacturing industry and have played significant roles in promoting the three types of patents. The negative effects of the LCC on the patent applications of listed companies have been manifested mainly in the suppression of invention patent applications by listed non-manufacturing companies. On the whole, the LCC has not significantly affected the number of patent applications by listed companies.

#### 4.5.3. Effects of Resource Mismatches

Studies have found that the mechanism for improving the efficiency of resource allocation among enterprises and promoting TFP lies in the flow of production factors from low-to high-productivity firms [54]. The carbon emission requirements and target assessments of the pilot low-carbon cities have encouraged more production factors to be invested in the state-owned sector or the low-productivity, low-carbon, and environment-friendly industries, resulting in a deepening of resource misallocation and hindering the improvement of technical efficiency [55].

Following Duranton et al. [56], as well as Olley and Pakes [57], we used data from the Chinese Industrial Enterprise Database to measure the efficiency of resource allocation with the covariance between the enterprise factor share and the productivity of each city (OP covariance) as an indicator of the efficiency of the resource allocation in an industry. Furthermore, we used the proportion of the labor force in each industry as the weight and summed up its resource allocation efficiency to calculate the resource allocation efficiency (RA) of the prefecture-level cities. The data for the mechanism test were provided by the China Industrial Enterprise Database, CEIC, CSMAR, and Chinese Research Data Services Platform (CNRDS). Table 14 shows the regression estimations of the LCC on the resource allocation efficiency of the cities. The establishment of the pilot cities has produced a significant resource mismatch effect, which is reflected in the significantly negative estimated coefficients of the LCC after the addition of the time and region fixed effects, clustering standard errors at the city level, and control variables in sequence.

The results of the mechanism analysis may have been due to three causes. First, the LCC may have promoted the scale efficiency of the cities through the upgrading of the industrial structure. Second, the LCC may have accelerated the technological innovation of the cities. On the macro-level, the LCC has promoted patent applications in the whole city. On the micro-level, it has promoted the patent applications by listed manufacturing companies, for which the invention, utility model, and design patents have been significantly and positively affected. However, no evidence suggests that the LCC has promoted the technological innovation of the listed non-manufacturing companies. Finally, the LCC may have led to serious resource mismatches among enterprises, thus inhibiting the improvement of technical efficiency.

## 5. Discussion

We found that the LCC in China has positively affected TFP, which is similar to the findings of Qi et al. [58], whose study of the carbon emission trading policy in China found that its reduction of carbon emissions and energy consumption had not been at the expense of economic development. The mechanism analysis showed that the LCC has affected TFP mainly through three channels: the scale efficiency, technical progress, and technical efficiency of urban production.

The preferential policies in the pilot cities have played significant roles and created the conditions for the implementation of various government policy tools and incentives while promoting the development of industrial scale and specialization. With the in-depth implementation of the pilot policy, the production costs of some high-polluting enterprises have continued to rise, prompting them to change their development models or exit the market directly [13]. The energy-saving and environmentally-friendly enterprises in the secondary and tertiary industries have gradually gained development advantages, thereby promoting the upgrading of the industrial structure [11]. The optimization and rationalization of the industrial structure have continued to deepen the division of labor in the production process and new departments have continued to emerge. As a result of the division of labor, the specialization of different links in the production process has strengthened. Workers accumulate specialized knowledge and skills, thus realizing industrial scale, gradually increasing returns to scale, achieving economies of scale, and increasing scale efficiency.

The pilot low-carbon cities have been committed to improving individual innovation capabilities and environmental quality without affecting economic development. Therefore, technical progress is the second path by which the LCC can increase TFP. First, environmental regulations promote technological R&D and green innovation by enterprises [59]. The government’s assessments of the carbon emission targets and production costs will force high-carbon companies, especially those in manufacturing, to improve their innovation capabilities, as well as to adopt new equipment and new technologies, thus promoting green innovation. Second, to achieve the goal of low-carbon development, the industries have evolved successively from being labor-intensive to capital-intensive and then technology-intensive. This evolution has provided a continuous and huge market demand for the application of new technologies while the upgrading of industrial structure has necessitated technological innovation. Moreover, the upgrading has reduced the proportions of the heavy and high-carbon industries [11], allowing those with high levels of innovation to gradually replace those with lower levels, thus increasing the overall level of technological innovation. Finally, to balance environmental protection and economic development, the government has subsidized some enterprises with innovative potential in order to encourage investments in scientific and technological R&D, increase the introduction of high-quality talents, and promote the creation of technological progress in the cities, through the flow of human capital and through knowledge spillover effects.

The effects of the LCC on technical efficiency are also worthy of attention. On the one hand, the policies of the central government are often poorly implemented or distorted by local government officials [60]. To meet the low-carbon assessment standards, some local governments use ecological and environmental protection as an excuse to blindly expand investments and constructions in disguised enclosures. These acts not only distort the decisive role of the market in resource allocation but also inhibit the enthusiasm and initiative of enterprises to gather spontaneously, thus leading to serious resource misallocation. The excessive concentration of resources in some enterprises affects the efficiency of resource allocation and is not conducive to the transformation of innovation achievements or the improvement of the technical efficiency of the cities [61]. On the other hand, policy preferences and specific goals for the establishment of low-carbon environmental protection industries in the pilot cities have been supported by local governments. However, subsidized competition among regions has led to the excessive concentration of resources in certain areas, which affects the locating of companies and their internal linkages, thus inhibiting the synergies of industries. The material and human capital in the region are overly concentrated in enterprises with low energy consumption, low emissions, and low pollution. Enterprises with higher productivity do not receive sufficient factor inputs, causing resource misallocation, which, in turn, affects overall production efficiency [62].

## 6. Conclusions and Policy Implications

This study focused on the effects of low-carbon city construction (LCC) on the total factor productivity (TFP) of prefecture-level cities in China, then explored the mechanism of how the LCC affected TFP and its components. In this section, we present some suggestions for the development of low-carbon cities. LCC has significantly and positively affected TFP, but the effects on each component have been different. The policy for the construction of pilot low-carbon cities has promoted scale efficiency and technical progress but inhibited the technical efficiency of the cities. Regional heterogeneity analysis showed that the policy has significantly affected TFP more in the eastern than in the central and western regions. In the eastern region, the LCC has promoted technical progress but has not significantly affected scale and technical efficiency. In the central and western regions, the LCC has promoted technical progress and scale efficiency but has hindered technical efficiency. To explain these results, we have postulated three paths for the mechanism by which the LCC affects TFP. Our mechanism analysis showed that resource mismatches among enterprises have been the main reason for reduced technical efficiency, whereas technological innovation and the upgrading of the industrial structure have promoted technical progress and scale efficiency. The analysis in this article is for China, but the conclusions and policy recommendations of this article also have reference value and significance for other developing countries. Considering the differences of different countries, future research could be carried out based on other countries.

The LCC in China has had complex effects on TFP. Although it has promoted the TFP of cities in general, technical efficiency still faces severe challenges. The policy implications are discussed below.

First, the baseline regression showed that the LCC had significantly improved the TFP of the cities. Building low-carbon cities is an important method commonly used in developed countries to improve air quality. For China, a developing country, pilot low-carbon cities have also played important roles in reducing carbon emissions, promoting technological innovation, and improving production efficiency. Therefore, the good use of the policy carrier of the pilot low-carbon cities to extend the low-carbon development model to the whole country is necessary.

Second, the regional heterogeneity analysis showed significant regional differences in the effects of the policy. Therefore, a differentiated development plan with targeted measures that incorporate the geographical location, resource endowment, environmental quality, and current economic and social development of each region should be formulated. Production should be developed according to local conditions. Full use of the factor endowments and comparative advantages of various regions is necessary. A low-carbon development model that is conducive to environmental quality improvement and productivity growth is necessary to realize the sustainable development of China’s economy.

Third, the results of the mechanism analysis showed that the main effects of the pilot policy were due to the innovation behaviors of enterprises and the upgrading of industrial structure. The efficiency of regional resource allocation requires improvement. Urban construction is expected to continue encouraging the system and mechanism supporting basic research and innovation while improving projects for the cultivation of scientific and technological talents, as well as for industrial upgrading. Moreover, we should pay more attention to the transformation of traditional industries to low-carbon environmental protection and emerging industries, promote industrial integration and coordinated development, strengthen the correlation between enterprises through factor flow and information exchange, and allocate to enterprises more production factors in order to achieve the goals of energy conservation, environmental protection, and economic development while improving the efficiency of resource allocation and overall technical efficiency.

## Figures and Tables

**Table 1:** Primary variables and processing methods.

Variable Type	Symbol	Variable Name	Processing Methods
Dependent variable	TFP	Total factor productivity	Stochastic frontier analysis (SFA)
Independent variable	Policy	Pilot program for low-carbon cities	Dummy variable
Macro-control variable	IND	Industrial structure	(Added value of secondary industry in the region/regional GDP) × 100%
	ECO	Level of economic development	Regional GDP/total population
	OPEN	Degree of openness	(The actual FDI in the region/regional GDP) × 100%
	DEN	Population density	Regional total population at the end of the year/administrative area
	GOV	Government size	(Budgeted government expenditures/regional GDP) × 100%
	AUT	Financial autonomy	Budgeted government revenue/budgeted expenditures
	GRD	Government research and development (R&D) funding intensity	(Government expenditures on science + expenditures on education)/budgeted expenditures
Enterprise control variable	SIZE	Enterprise scale	Total assets of the enterprise
	FIN	Financial situation	Total liabilities/total assets of the enterprise
	ROA	Return on assets	Net profit/total assets of the enterprise
	INT	Proportion of intangible assets	Net intangible assets/total assets of the enterprise
	SUB	Government subsidy intensity	Government subsidies/operating income of the enterprise

**Table 2:** Descriptive statistics.

Variable Type	Symbol	Sample Size	Mean	Standard Deviation	Min.	Max.
Dependent variable	TFP	2208	0.8853	0.0344	0.7848	1.0112
Independent variable	Policy	2208	0.0399	0.1958	0.0000	1.0000
Macro-control variable	IND	2208	0.4583	0.1339	0.0862	0.8440
	ECO	2208	10.2786	0.7782	7.3063	13.1084
	OPEN	2208	0.0223	0.0285	0.0000	1.0124
	DEN	2208	5.8594	0.8150	1.7404	7.8400
	GOV	2208	0.1562	0.1443	0.0427	6.3884
	AUT	2208	0.5085	0.2294	0.0400	1.5413
	GRD	2208	0.2017	0.0455	0.0158	0.3868
Enterprise control variable	SIZE	10,268	21.7701	1.2115	15.5773	28.0035
	FIN	10,268	0.3473	0.1524	0.0071	0.8248
	ROA	10,268	0.0398	0.0593	−1.0773	1.0958
	INT	10,268	0.0464	0.0537	0.0000	0.6098
	SUB	10,268	2.8024	0.4118	0.0000	3.2902

TFP: Total factor productivity; Policy: Pilot program for low-carbon cities; IND: Industrial structure; ECO: Level of economic development; OPEN: Degree of openness; DEN: Population density; GOV: Government size; AUT: Financial autonomy; GRD: Government R&D funding intensity; SIZE: Enterprise scale; FIN: Financial situation; ROA: Return on assets; INT: Proportion of intangible assets; SUB: Government subsidy intensity.

**Table 3:** Effects of low-carbon city construction (LCC) on the total factor productivity (TFP).

Variable	TFP								
	(1)	(2)	(3)	(4)	(5)	(6)	(7)	(8)	(9)
Policy	0.049 *	0.074 **	0.075 **	0.072 **	0.074 **	0.074 **	0.074 **	0.068 **	0.069 **
	(1.68)	(2.43)	(2.45)	(2.39)	(2.45)	(2.46)	(2.45)	(2.29)	(1.97)
IND		0.627 ***	0.638 ***	0.641 ***	0.674 ***	0.677 ***	0.677 ***	0.663 ***	0.748 ***
		(3.38)	(3.48)	(3.51)	(3.71)	(3.72)	(3.70)	(3.72)	(3.84)
ECO			−0.060	−0.098	−0.096	−0.108	−0.109	−0.067	−0.052
			(−1.50)	(−1.56)	(−1.51)	(−1.54)	(−1.53)	(−0.92)	(−0.61)
OPEN				−0.348 ***	−0.334 ***	−0.159 ***	−0.171 ***	−0.289 ***	−0.186 ***
				(−3.30)	(−3.24)	(−4.37)	(−4.38)	(−5.64)	(−3.35)
DEN					0.061	0.062	0.062	0.086	−0.042
					(0.36)	(0.37)	(0.36)	(0.52)	(−0.23)
GOV						−0.043 ***	−0.042 *	0.015	0.009
						(−2.75)	(−1.73)	(1.18)	(1.10)
AUT							0.008 **	0.007	0.014
							(2.08)	(1.07)	(1.13)
GRD								0.736 **	0.715 ***
								(2.12)	(3.81)
Regional fixed effect	Yes	Yes	Yes	Yes	Yes	Yes	Yes	Yes	Yes
Year fixed effect	Yes	Yes	Yes	Yes	Yes	Yes	Yes	Yes	Yes
_cons	−1.005 ***	−1.279 ***	−0.708 ***	−0.333 **	−0.722 **	−0.618 **	−0.610 **	−1.281 **	−0.723 **
	(−6.59)	(−5.72)	(−5.80)	(−2.54)	(−2.36)	(−2.17)	(−2.30)	(−2.35)	(−2.29)
Observations	2208	2208	2208	2208	2208	2208	2208	2208	2160
Adj. R-squared	0.202	0.210	0.210	0.210	0.211	0.211	0.211	0.213	0.209

Note: *t*-values are reported in parentheses. The robust standard errors are clustered at the city level. ***, **, and * represent significance at the levels of 1%, 5%, and 10%, respectively. TFP: Total factor productivity; Policy: Pilot program for low-carbon cities; IND: Industrial structure; ECO: Level of economic development; OPEN: Degree of openness; DEN: Population density; GOV: Government size; AUT: Financial autonomy; GRD: Government R&D funding intensity; SIZE: Enterprise scale; FIN: Financial situation; ROA: Return on assets; INT: Proportion of intangible assets; SUB: Government subsidy intensity; _cons: constant term; Adj.: adjusted.

**Table 4:** Effects of low-carbon city construction (LCC) on the components of the total factor productivity (TFP).

Variable	Scale Efficiency		Technical Progress		Technical Efficiency	
	(1)	(2)	(3)	(4)	(5)	(6)
Policy	0.151 ***	0.212 ***	0.133 ***	0.130 **	−0.045 **	−0.044 ***
	(4.29)	(5.88)	(3.43)	(2.39)	(−2.61)	(−2.94)
IND		0.563 ***		0.100 *		0.153 *
		(3.73)		(1.73)		(1.71)
ECO		0.056		−0.111 ***		−0.062
		(0.79)		(−4.95)		(−1.04)
OPEN		1.337 **		−0.461 ***		−1.539 ***
		(2.31)		(−4.42)		(−3.49)
DEN		−0.159		−0.045		0.113
		(−0.92)		(−0.60)		(1.13)
GOV		−0.153		0.008		0.189 **
		(−1.42)		(0.41)		(2.36)
AUT		−0.045		−0.001		0.088
		(−0.41)		(−0.03)		(1.37)
GRD		0.147 ***		0.199 ***		0.187 ***
		(2.41)		(3.17)		(2.02)
Regional fixed effect	Yes	Yes	Yes	Yes	Yes	Yes
Year fixed effect	Yes	Yes	Yes	Yes	Yes	Yes
_cons	0.124 ***	0.312 ***	−1.087 ***	0.159 ***	−0.012 ***	−0.213 ***
	(6.45)	(5.22)	(−26.94)	(10.32)	(−3.39)	(−3.23)
Observations	2208	2208	2208	2208	2208	2208
Adj. R-squared	0.012	0.017	0.383	0.494	0.108	0.119

Note: *t*-values are reported in parentheses. The robust standard errors are clustered at the city level. ***, **, and * represent significance at the levels of 1%, 5%, and 10%, respectively. TFP: Total factor productivity; Policy: Pilot program for low-carbon cities; IND: Industrial structure; ECO: Level of economic development; OPEN: Degree of openness; DEN: Population density; GOV: Government size; AUT: Financial autonomy; GRD: Government R&D funding intensity; SIZE: Enterprise scale; FIN: Financial situation; ROA: Return on assets; INT: Proportion of intangible assets; SUB: Government subsidy intensity; _cons: constant term; Adj.: adjusted.

**Table 5:** Placebo test.

Variable	TFP	Scale Efficiency	Technical Progress	Technical Efficiency	TFP	Scale Efficiency	Technical Progress	Technical Efficiency
	(1)	(2)	(3)	(4)	(5)	(6)	(7)	(8)
Policy-advance1	0.042	0.037	0.007	−0.018				
	(1.48)	(1.06)	(0.90)	(−0.70)				
Policy-advance2					0.014	−0.006	0.011	0.000
					(0.49)	(−0.16)	(1.45)	(0.00)
Control	Yes	Yes	Yes	Yes	Yes	Yes	Yes	Yes
Regional fixed effect	Yes	Yes	Yes	Yes	Yes	Yes	Yes	Yes
Year fixed effect	Yes	Yes	Yes	Yes	Yes	Yes	Yes	Yes
_cons	−1.779 ***	0.460 **	−0.602 **	−1.960 ***	0.975 ***	3.045 **	−0.531 ***	−2.056 ***
	(−4.01)	(2.43)	(−2.60)	(−3.79)	(3.52)	(2.53)	(−4.33)	(−4.89)
Observations	2024	2024	2024	2024	1840	1840	1840	1840
Adj. R-squared	0.167	0.021	0.913	0.151	0.116	0.020	0.907	0.167

Note: *t*-values are reported in parentheses. The robust standard errors are clustered at the city level. *** and ** represent significance at the levels of 1% and 5% respectively. TFP: Total factor productivity; Policy: Pilot program for low-carbon cities; _cons: constant term; Adj.: adjusted.

**Table 6:** Reselected experimental and control groups.

Variable	TFP		Scale Efficiency		Technical Progress		Technical Efficiency	
	(1)	(2)	(3)	(4)	(5)	(6)	(7)	(8)
Policy	0.045 **	0.061 *	0.168 **	0.202 ***	0.036 **	0.032 **	−0.054 ***	−0.063 ***
	(2.30)	(1.92)	(2.10)	(4.25)	(2.40)	(2.47)	(−5.35)	(−3.76)
Control	No	Yes	No	Yes	No	Yes	No	Yes
Regional fixed effect	Yes	Yes	Yes	Yes	Yes	Yes	Yes	Yes
Year fixed effect	Yes	Yes	Yes	Yes	Yes	Yes	Yes	Yes
_cons	−1.009 ***	−1.270 ***	0.179 ***	0.857 ***	−1.126 ***	−0.120 ***	−0.062 ***	−2.374 **
	(−10.94)	(−5.82)	(8.53)	(3.45)	(−8.86)	(−3.27)	(−5.28)	(−2.26)
Observations	3084	3084	3084	3084	3084	3084	3084	3084
Adj. R-squared	0.194	0.201	0.011	0.010	0.579	0.587	0.135	0.142

Note: *t*-values are reported in parentheses. The robust standard errors are clustered at the city level. ***, **, and * represent significance at the levels of 1%, 5%, and 10%, respectively. TFP: Total factor productivity; Policy: Pilot program for low-carbon cities; _cons: constant term; Adj.: adjusted.

**Table 7:** Single-difference test.

Variable	TFP		Scale Efficiency		Technical Progress		Technical Efficiency	
	(1)	(2)	(3)	(4)	(5)	(6)	(7)	(8)
Policy	−0.194 ***	−0.144 ***	0.215 **	0.332 **	−0.145 ***	−0.124 **	−0.095 ***	−0.087 ***
	(−7.37)	(−5.15)	(2.39)	(2.53)	(−2.99)	(−2.26)	(−8.77)	(−6.34)
Control	No	Yes	No	Yes	No	Yes	No	Yes
Regional fixed effect	Yes	Yes	Yes	Yes	Yes	Yes	Yes	Yes
Year fixed effect	No	No	No	No	No	No	No	No
_cons	−1.139 ***	4.934 ***	0.150 ***	1.546*	−1.294 ***	4.560 ***	1.021 *	−1.320 *
	(−10.51)	(3.32)	(12.74)	(1.79)	(−8.32)	(3.84)	(1.83)	(−1.74)
Observations	2208	2208	2208	2208	2208	2208	2208	2208
Adj. R-squared	0.008	0.047	0.001	0.004	0.040	0.300	0.002	0.017

Note: *t*-values are reported in parentheses. The robust standard errors are clustered at the city level. ***, **, and * represent significance at the levels of 1%, 5%, and 10%, respectively. TFP: Total factor productivity; Policy: Pilot program for low-carbon cities; _cons: constant term; Adj.: adjusted.

**Table 8:** Regional heterogeneity analysis.

Variable	East City				Midwest City			
	TFP	Scale Efficiency	Technical Progress	Technical Efficiency	TFP	Scale Efficiency	Technical Progress	Technical Efficiency
	(1)	(2)	(3)	(4)	(5)	(6)	(7)	(8)
Policy	0.010 **	0.007	0.005 ***	−0.000	−0.001	0.010 ***	0.004 ***	−0.015 ***
	(2.41)	(1.37)	(3.35)	(−0.09)	(−0.15)	(2.70)	(7.78)	(−6.39)
Control	Yes	Yes	Yes	Yes	Yes	Yes	Yes	Yes
Regional fixed effect	Yes	Yes	Yes	Yes	Yes	Yes	Yes	Yes
Year fixed effect	Yes	Yes	Yes	Yes	Yes	Yes	Yes	Yes
_cons	−0.172 **	0.100 ***	−0.098 *	−0.200 *	−0.192 ***	0.214 ***	−0.123 ***	−0.338 ***
	(−3.74)	(3.35)	(−1.98)	(−1.70)	(−3.01)	(5.93)	(−3.76)	(−2.82)
Observations	864	864	864	864	1344	1344	1344	1344
Adj. R-squared	0.184	0.023	0.870	0.168	0.220	0.013	0.903	0.147

Note: *t*-values are reported in parentheses. The robust standard errors are clustered at the city level. ***, **, and * represent significance at the levels of 1%, 5%, and 10%, respectively. TFP: Total factor productivity; Policy: Pilot program for low-carbon cities; _cons: constant term; Adj.: adjusted.

**Table 9:** Effects of low-carbon city construction (LCC) on upgrading of industrial structure.

Variable	TS				TL			
	(1)	(2)	(3)	(4)	(5)	(6)	(7)	(8)
Policy	0.106 ***	0.098 ***	0.098 **	0.068 *	−0.028	−0.028	−0.028 ***	−0.028 ***
	(5.85)	(5.36)	(2.24)	(1.91)	(−0.64)	(−0.64)	(−4.68)	(−3.34)
Control	No	No	No	Yes	No	No	No	Yes
Regional fixed effect	No	Yes	Yes	Yes	No	Yes	Yes	Yes
Year fixed effect	Yes	Yes	Yes	Yes	Yes	Yes	Yes	Yes
Clustering at the city level	No	No	Yes	Yes	No	No	Yes	Yes
_cons	0.819 ***	0.824 ***	0.824 ***	5.142 ***	0.001 *	0.252 ***	0.252 ***	1.802 ***
	(8.51)	(9.63)	(7.42)	(3.81)	(1.71)	(9.18)	(6.29)	(3.12)
Observations	2208	2208	2208	2208	2208	2208	2208	2208
Adj. R-squared	0.050	0.089	0.184	0.350	0.076	0.044	0.066	0.079

Note: *t*-values are reported in parentheses. The robust standard errors are clustered at the city level. ***, **, and * represent significance at the levels of 1%, 5%, and 10%, respectively. Policy: Pilot program for low-carbon cities; TS: industrial structure optimization; TL: industrial structure rationalization; _cons: constant term; Adj.: adjusted.

**Table 10:** Effects of low-carbon city construction (LCC) on technological innovation of cities.

Variable	Innovation		Invention		Utility Model		Design	
	(1)	(2)	(3)	(4)	(5)	(6)	(7)	(8)
Policy	0.155 ***	0.143 **	0.237 **	0.198 **	0.169 ***	0.146 ***	0.125 *	0.131 *
	(2.71)	(2.43)	(2.41)	(2.07)	(2.94)	(2.74)	(1.70)	(1.73)
Control	No	Yes	No	Yes	No	Yes	No	Yes
Regional fixed effect	Yes	Yes	Yes	Yes	Yes	Yes	Yes	Yes
Year fixed effect	Yes	Yes	Yes	Yes	Yes	Yes	Yes	Yes
Clustering at the city level	Yes	Yes	Yes	Yes	Yes	Yes	Yes	Yes
_cons	0.829 ***	1.059 **	0.319 ***	3.915 **	0.515 ***	5.377 ***	0.375 ***	−3.693 **
	(11.14)	(2.41)	(15.26)	(2.45)	(6.78)	(2.64)	(12.70)	(−2.10)
Observations	2208	2208	2208	2208	2208	2208	2208	2208
Adj. R-squared	0.774	0.783	0.602	0.631	0.705	0.725	0.381	0.407

Note: *t*-values are reported in parentheses. The robust standard errors are clustered at the city level. ***, **, and * represent significance at the levels of 1%, 5%, and 10%, respectively. Policy: Pilot program for low-carbon cities; _cons: constant term; Adj.: adjusted.

**Table 11:** Effects of low-carbon city construction (LCC) on technological innovation of listed companies.

Variable	Full Sample		Manufacturing		Non-Manufacturing	
	(1)	(2)	(3)	(4)	(5)	(6)
Policy	0.031	0.020	0.237 ***	0.224 ***	−0.065 *	−0.084 **
	(1.09)	(0.68)	(9.09)	(7.28)	(−1.83)	(−2.09)
Control	No	Yes	No	Yes	No	Yes
Regional fixed effect	Yes	Yes	Yes	Yes	Yes	Yes
Year fixed effect	Yes	Yes	Yes	Yes	Yes	Yes
Clustering at the city level	Yes	Yes	Yes	Yes	Yes	Yes
_cons	1.042 ***	3.848 ***	0.169 ***	5.325 ***	2.317 ***	−1.767 ***
	(9.36)	(8.05)	(8.91)	(3.57)	(4.33)	(−5.45)
Observations	10268	10268	4625	4625	5643	5643
Adj. R-squared	0.051	0.055	0.040	0.050	0.063	0.071

Note: *t*-values are reported in parentheses. The robust standard errors are clustered at the city level. ***, **, and * represent significance at the levels of 1%, 5%, and 10%, respectively; _cons: constant term; Adj.: adjusted.

**Table 12:** Effects of low-carbon city construction (LCC) on research and development (R&D) expenditures of listed companies.

Variable	Full Sample		Manufacturing		Non-Manufacturing	
	(1)	(2)	(3)	(4)	(5)	(6)
Policy	0.031	0.045	0.084 **	0.039 **	−0.062 *	−0.067 *
	(1.15)	(1.38)	(2.51)	(2.24)	(−1.70)	(−1.75)
Control	No	Yes	No	Yes	No	Yes
Regional fixed effect	Yes	Yes	Yes	Yes	Yes	Yes
Year fixed effect	Yes	Yes	Yes	Yes	Yes	Yes
Clustering at the city level	Yes	Yes	Yes	Yes	Yes	Yes
_cons	15.966 ***	2.044 ***	15.678 ***	−0.951 ***	16.229 ***	2.730 ***
	(7.97)	(7.41)	(9.53)	(−4.10)	(10.26)	(3.58)
Observations	10268	10268	4625	4625	5643	5643
Adj. R-squared	0.408	0.438	0.357	0.406	0.433	0.457

Note: *t*-values are reported in parentheses. The robust standard errors are clustered at the city level. ***, **, and * represent significance at the levels of 1%, 5%, and 10%, respectively. Policy: Pilot program for low-carbon cities; _cons: constant term; Adj.: adjusted.

**Table 13:** Effects of low-carbon city construction (LCC) on various types of patent applications of listed companies.

Variable	Full Sample			Manufacturing			Non-Manufacturing		
	Invention	Utility Model	Design	Invention	Utility Model	Design	Invention	Utility Model	Design
	(1)	(2)	(3)	(4)	(5)	(6)	(7)	(8)	(9)
Policy	−0.102 ***	0.052 **	0.030 *	0.093 ***	0.097 ***	0.023 ***	−0.111 **	−0.007	0.070 *
	(−3.91)	(2.11)	(1.69)	(4.12)	(4.91)	(3.74)	(−2.53)	(−0.15)	(1.87)
Control	Yes	Yes	Yes	Yes	Yes	Yes	Yes	Yes	Yes
Regional fixed effect	Yes	Yes	Yes	Yes	Yes	Yes	Yes	Yes	Yes
Year fixed effect	Yes	Yes	Yes	Yes	Yes	Yes	Yes	Yes	Yes
Clustering at the city level	Yes	Yes	Yes	Yes	Yes	Yes	Yes	Yes	Yes
_cons	5.467 ***	3.332 ***	−0.718 **	2.991 ***	3.825 **	−0.297 ***	−2.111 ***	−5.124 ***	−4.380 **
	(3.83)	(2.93)	(−2.43)	(5.24)	(2.45)	(−2.96)	(−5.45)	(−3.40)	(−2.41)
Observations	10268	10268	10268	4625	4625	4625	5643	5643	5643
Adj. R-squared	0.079	0.072	0.008	0.049	0.041	0.012	0.108	0.098	0.017

Note: *t*-values are reported in parentheses. The robust standard errors are clustered at the city level. ***, **, and * represent significance at the levels of 1%, 5%, and 10%, respectively. Policy: Pilot program for low-carbon cities; _cons: constant term; Adj.: adjusted.

**Table 14:** Effects of low-carbon city construction (LCC) on resource allocation efficiency.

Variable	RA			
	(1)	(2)	(3)	(4)
Policy	−0.779 **	−0.909 **	−0.909 ***	−0.740 **
	(−2.24)	(−2.43)	(−2.87)	(−2.50)
Control	No	No	No	Yes
Regional fixed effect	No	Yes	Yes	Yes
Year fixed effect	Yes	Yes	Yes	Yes
Clustering at the city level	No	No	Yes	Yes
_cons	0.081 **	0.082 ***	0.082 **	−2.445 ***
	(2.36)	(3.51)	(2.55)	(−3.77)
Observations	2208	2208	2208	2208
Adj. R-squared	0.051	0.064	0.059	0.071

Note: *t*-values are reported in parentheses. The robust standard errors are clustered at the city level. *** and ** represent significance at the levels of 1% and 5% respectively. Policy: Pilot program for low-carbon cities; _cons: constant term; Adj.: adjusted.

## Data Availability

Not applicable.

## References

[1] Yang F., Shi B., Xu M., Feng C. (2019). Can reducing carbon emissions improve economic performance–evidence from China. Economics.

[2] Matus K., Nam K.M., Selin N.E., Lamsal L.N., Reilly J.M., Paltsev S. (2012). Health damages from air pollution in China. Glob. Environ. Chang..

[3] Cheng J., Yi J., Dai S., Xiong Y. (2019). Can low-carbon city construction facilitate green growth? Evidence from China’s pilot low-carbon city initiative. J. Clean. Prod..

[4] Environmental Performance Index (2020). Yale Center for Environmental Law & Policy, New Haven. https://epi.yale.edu/.

[5] Friedlingstein P., Jones M.W., O’sullivan M., Andrew R.M., Hauck J. (2019). Global carbon budget 2019. Earth Syst. Sci. Data.

[6] Huang R., Zhang Y., Bozzetti C., Ho K., Cao J., Han Y., Daellenbach K.R., Slowik J.G., Platt S.M., Prévôt A.S. (2014). High secondary aerosol contribution to particulate pollution during haze events in China. Nature.

[7] Yuan X., Zuo J. (2011). Transition to low carbon energy policies in China—From the Five-Year Plan perspective. Energy Policy.

[8] Wolff H. (2014). Keep your clunker in the suburb: Low-emission zones and adoption of green vehicles. Econ. J..

[9] Fu Y., He C., Luo L. (2021). Does the low-carbon city policy make a difference? Empirical evidence of the pilot scheme in China with DEA and PSM-DID. Ecol. Indic..

[10] Liu Z., Guan D., Crawford-Brown D., Zhang Q., He K., Liu J. (2013). A low-carbon road map for China. Nature.

[11] Zheng J., Shao X., Liu W., Kong J., Zuo G. (2021). The impact of the pilot program on industrial structure upgrading in low-carbon cities. J. Clean. Prod..

[12] Bardaka I., Bournakis I., Kaplanoglou G. (2021). Total factor productivity (TFP) and fiscal consolidation: How harmful is austerity?. Econ. Model..

[13] Qiu S., Wang Z., Liu S. (2021). The policy outcomes of low-carbon city construction on urban green development: Evidence from a quasi-natural experiment conducted in China. Sustain. Cities Soc..

[14] Chen H., Guo W., Feng X., Wei W., Liu H., Feng Y., Gong W. (2021). The impact of low-carbon city pilot policy on the total factor productivity of listed enterprises in China. Resour. Conserv. Recycl..

[15] Solow R.M. (1957). Technical change and the aggregate production function. Rev. Econ. Stat..

[16] Wang H., Cui H., Zhao Q. (2021). Effect of green technology innovation on green total factor productivity in China: Evidence from spatial durbin model analysis. J. Clean. Prod..

[17] Feng C., Huang J.B., Wang M. (2018). Analysis of green total-factor productivity in China’s regional metal industry: A meta-frontier approach. Resour. Policy.

[18] Dettori B., Marrocu E., Paci R. (2012). Total factor productivity, intangible assets and spatial dependence in the European regions. Reg. Stud..

[19] Siller M., Schatzer T., Walde J., Tappeiner G. (2021). What drives total factor productivity growth? An examination of spillover effects. Reg. Stud..

[20] Thota N., Subrahmanyam A.C.V. (2020). Bank total factor productivity convergence: Evidence from India. Finance Res. Lett..

[21] Tang H., Liu J., Wu J. (2020). The impact of command-and-control environmental regulation on enterprise total factor productivity: A quasi-natural experiment based on China’s “Two Control Zone” policy. J. Clean. Prod..

[22] Zhang W., Meng J., Tian X. (2020). Does de-capacity policy enhance the total factor productivity of China’s coal companies? A Regression Discontinuity design. Resour. Policy.

[23] Markowska-Przybyła U. (2020). Does Social Capital Matter for Total Factor Productivity? Exploratory Evidence from Poland. Sustainability.

[24] Lopez-Rodriguez J., Martinez-Lopez D. (2017). Looking beyond the R&D effects on innovation: The contribution of non-R&D activities to total factor productivity growth in the EU. Struct. Chang. Econ. D.

[25] Xiao Z., Peng H., Pan Z. (2021). Innovation, external technological environment and the total factor productivity of enterprises. Acc. Financ..

[26] Herzer D. (2011). The long-run relationship between outward foreign direct investment and total factor productivity: Evidence for developing countries. J. Dev. Stud..

[27] Miao J., Wang P. (2012). Bubbles and total factor productivity. Am. Econ. Rev..

[28] Furceri D., Celik S.K., Jalles J.T., Koloskova K. (2021). Recessions and total factor productivity: Evidence from sectoral data. Econ. Model..

[29] Chay K.Y., Greenstone M. (2003). Air Quality, Infant Mortality, and the Clean Air Act of 1970.

[30] Hand J.L., Prenni A.J., Copeland S., Schichtel B.A., Malm W.C. (2020). Thirty years of the Clean Air Act Amendments: Impacts on haze in remote regions of the United States (1990–2018). Atmos. Environ..

[31] Greenstone M., Hanna R. (2014). Environmental regulations, air and water pollution, and infant mortality in India. Am. Econ. Rev..

[32] Tanaka S. (2015). Environmental regulations on air pollution in China and their impact on infant mortality. J. Health Econ..

[33] Feng Y., Wang X., Liang Z., Hu S., Xie Y., Wu G. (2021). Effects of emission trading system on green total factor productivity in China: Empirical evidence from a quasi-natural experiment. J. Clean. Prod..

[34] Hanna R.N., Oliva P. (2010). The impact of inspections on plant-level air emissions. BE J. Econ. Anal. Policy.

[35] Chang T., Graff Zivin J., Gross T., Neidell M. (2016). Particulate pollution and the productivity of pear packers. Am. Econ. J. Econ. Policy.

[36] Ellison R.B., Greaves S.P., Hensher D.A. (2013). Five years of London’s low emission zone: Effects on vehicle fleet composition and air quality. Transport. Res. Part D Transp. Environ..

[37] Gehrsitz M. (2017). The effect of low emission zones on air pollution and infant health. J. Environ. Econ. Manag..

[38] Yu Y., Zhang N. (2021). Low-carbon city pilot and carbon emission efficiency: Quasi-experimental evidence from China. Energy Econ..

[39] Phdungsilp A. (2010). Integrated energy and carbon modeling with a decision support system: Policy scenarios for low-carbon city development in Bangkok. Energy Policy.

[40] Li L., Chen C., Xie S., Huang C., Cheng Z., Wang H., Wang Y., Huang H., Lu J., Dhakal S. (2010). Energy demand and carbon emissions under different development scenarios for Shanghai, China. Energy Policy.

[41] Yu Y., Wang Z., Liu D., Fu L. (2019). Changes in officials, total factor productivity fluctuation and government transformation: Evidence from Chinese prefecture cities. Pac. Econ. Rev..

[42] Kumbhakar S., Lovell C. (2000). Stochastic Frontier Analysis.

[43] Zhang J. (2008). Estimation of China’s provincial capital stock (1952–2004) with applications. J. Chin. Econ. Bus. Stud..

[44] Gao Y., Zhang M., Zheng J. (2021). Accounting and determinants analysis of China’s provincial total factor productivity considering carbon emissions. Chin. Econ. Rev..

[45] Furman J.L., Porter M.E., Stern S. (2002). The determinants of national innovative capacity. Resour. Policy.

[46] Liu D., Chen T., Liu X., Yu Y. (2019). Do more subsidies promote greater innovation? Evidence from the Chinese electronic manufacturing industry. Econ. Model..

[47] Beck T., Levine R., Levkov A. (2010). Big bad banks? The winners and losers from bank deregulation in the United States. J. Financ..

[48] Fan Z., Tian B. (2013). Tax competition, tax enforcement and tax avoidance. Econ. Res. J..

[49] Xin B., Qu Y. (2019). Effects of Smart City Policies on Green Total Factor Productivity: Evidence from a Quasi-Natural Experiment in China. Int. J. Environ. Res. Public Health.

[50] Liu R., Zhao R. (2015). Does the national high-tech zone promote regional economic development? A verification based on differences-in-differences method. Manag. World.

[51] Wang S., Fang C., Guan X., Pang B., Ma H. (2014). Urbanisation, energy consumption, and carbon dioxide emissions in China: A panel data analysis of China’s provinces. Appl. Energy.

[52] Gan C.H., Zheng R.G., Yu D.F. (2011). An Empirical Study on the Effects of Industrial Structure on Economic Growth and Fluctuations in China. Econ. Res. J..

[53] Wang K., Wu M., Sun Y., Shi X., Sun A., Zhang P. (2019). Resource abundance, industrial structure, and regional carbon emissions efficiency in China. Resour. Policy.

[54] Bartelsman E., Haltiwanger J., Scarpetta S. (2013). Cross-country differences in productivity: The role of allocation and selection. Am. Econ. Rev..

[55] Brandt L., Tombe T., Zhu X. (2013). Factor market distortions across time, space and sectors in China. Rev. Econ. Dynam..

[56] Duranton G., Ghani S.E., Goswami A.G., Kerr W.R. (2015). The Misallocation of Land and Other Factors of Production in India.

[57] Olley G.S., Pakes A. (1996). The Dynamics of Productivity in the Telecommunications Equipment Industry. Econometrica.

[58] Qi S., Cheng S., Cui J. (2021). Environmental and economic effects of China’s carbon market pilots: Empirical evidence based on a DID model. J. Clean. Prod..

[59] Liu Y., Wang A., Wu Y. (2021). Environmental regulation and green innovation: Evidence from China’s new environmental protection law. J. Clean. Prod..

[60] Lo K. (2014). China’s low-carbon city initiatives: The implementation gap and the limits of the target responsibility system. Habitat Int..

[61] Cai W., Ye P. (2020). How does environmental regulation influence enterprises’ total factor productivity? A quasi-natural experiment based on China’s new environmental protection law. J. Clean. Prod..

[62] Jones C.L. (2013). Misallocation, Economic Growth and Input-Output Economics.

